# Genome assembly of *Ramulariopsis gossypii*: a pathogen associated with areolate mildew in cotton (*Gossypium* spp.)

**DOI:** 10.1128/mra.00401-26

**Published:** 2026-06-29

**Authors:** Iago Beffart Schardong, Rachel Hill, Sejal Patel, Ankush Sharma, Alejandra M. Jimenez-Madrid, Jana Avant, Jenny Koebernick, Kaitlyn Bissonnette, Don C. Jones, Peng W. Chee

**Affiliations:** 1 Institute of Plant Breeding, Genetics and Genomics, University of Georgia 117299, Tifton, Georgia, USA; 2 Department of Crop, Soil and Environmental Sciences, Auburn University 1383, Auburn, Alabama, USA; 3 Department of Crop and Soil Sciences, The University of Georgia, Athens, Georgia, USA; 4 Department of Plant Pathology, University of Georgia, Tifton, Georgia, USA; 5 Department of Entomology and Plant Pathology, Auburn University 1383, Auburn, Alabama, USA; 6 Cotton Incorporated 2296 , Cary, North Carolina, USA; Rochester Institute of Technology, Rochester, New York, USA

**Keywords:** fungal pathogen, cotton, plant pathogens, whole genome sequencing

## Abstract

Areolate mildew is a foliar disease of cotton, with two fungal species reported as causal pathogens. We announce the first genome of *Ramulariopsis gossypii*. The genome size is 33.34 Mbp, with an N50 of 2.116 Mb and Benchmarking Universal Single-Copy Orthologs completeness of 98.8%, providing a resource for comparative genomics and disease management.

## ANNOUNCEMENT

Areolate mildew is a major cotton (*Gossypium* spp.) disease worldwide (1). In recent years, it has been associated with two pathogens, *Ramulariopsis pseudoglycines* and *Ramulariopsis gossypii*, which exhibit the same signs and symptoms leading to yield losses (2–4). Recently, a genome of *R. pseudoglycines* was published (5). To better understand the relation between the two causal species and their host, we sequenced and assembled a genome for *R. gossypii*.

In 2024, cotton plants at Auburn University (Alabama, USA) exhibited lesions on the adaxial leaf surface and white, powdery fungal sporulation on the abaxial surface. Symptomatic leaves were collected, and using a sterile dissecting probe, we transferred conidia aseptically directly from conidiophores to potato dextrose agar plates (39 g PDA/1 L deionized water amended with 1 mL lactic acid) and incubated in the dark at 25°C. After 14 days, grayish-white colonies with typical zero to three septate oblong spores, consistent with *Ramulariopsis* spp. (4, 6), were selected for single-spore isolation. Conidia were suspended in 0.01% Tween-20, serially diluted, and spread onto water agar. After incubation for 24–48 h at 25°C, individual germinating conidia were transferred to V8 agar plates (3 g/L of CaCO_3_, 200 mL/L of V8 juice, 17 g/L of agar, and 1 mL lactic acid) to obtain pure cultures. After confirmation of species identity using Sanger sequencing of the translation elongation factor-a and the internal transcribed spacer regions (1), a pure culture from a single colony was scraped, flash frozen, and stored at −80°C. Fungal samples were retrieved in 2026, and 1,000 µL of DNA/RNA Shield Stabilization Solution (Zymo) was added to sampling microtubes to maintain DNA integrity and inactivate the organism.

High molecular weight genomic DNA was extracted using the PacBio NanoBind PanDNA kit (PacBio, 103-260-000) following the manufacturer's recommendations for fungal samples. The DNA was eluted to a final volume of 50 µL, with concentrations determined by Qubit (Thermo Fisher Scientific). The DNA fragment size was evaluated via Agilent Femto Pulse analysis. Sequencing libraries were prepared using Revio SPRQ HiFi Prep Kit following the manufacturer’s protocol. Pooled libraries underwent the PacBio binding and cleanup steps using the Revio SPRQ Polymerase Kit. The prepared libraries were loaded on a Revio SMRT 25M Flowcell and sequenced using the PacBio Revio instrument. Demultiplexing, quality control, and adapter trimming were performed on instrument via Lima v2.13.0 (7).

**Fig 1:**
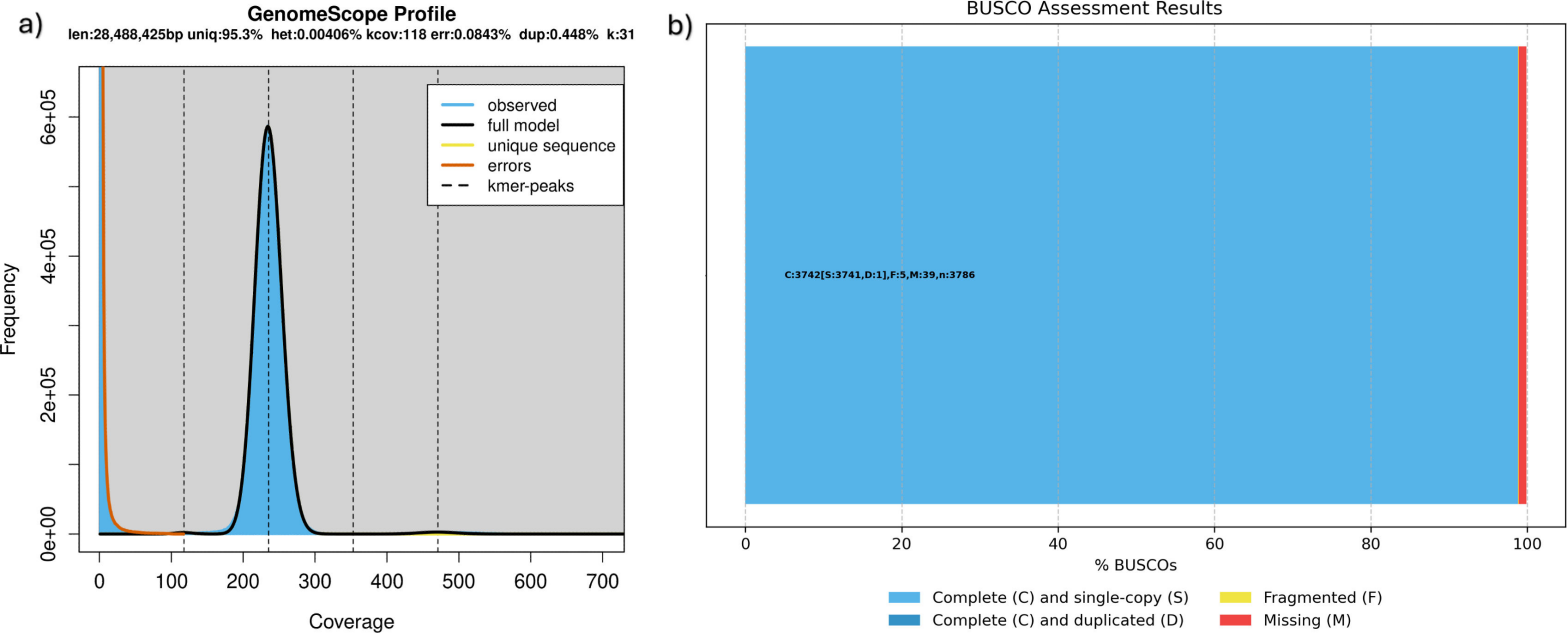
Genome size estimation and genome completeness assessment. (**a**) GenomeScope k-mer profile showing the distribution of k-mer coverage and model fit, estimating a genome size of ~28.49 Mb with 95.3% unique sequence, low heterozygosity, and minimal duplication. (**b**) BUSCO assessment indicating high completeness of the assembly, with most conserved single-copy orthologs recovered as complete and only a very small fraction fragmented or missing.

The sequenced data underwent a second quality control using LongQC v1.2.1 (8). The *de novo* genome assembly was completed using Flye v2.9.5 (9). Assembly quality was assessed using Quast v5.2.0 (10), and the completeness was assessed with Benchmarking Universal Single-Copy Orthologs (BUSCO) v6.0.0 using the dothideomycetes_odb10 database (Fig. 1) (11). Assembly coverage was assessed by aligning the PacBio library back to the assembled genome using BWA v0.7.19 (12, 13), and statistics were extracted using Qualimap v2.3 (14). Genome masking and repetitive regions were annotated using RepeatModeler v2.0.5 (15) and RepeatMasker v4.1.5 (16). Gene prediction and annotation were done in the soft-masked assembly using Funannotate v1.8.16 (17). All software was used with default parameters unless otherwise specified.

The total read count was 4,329,065 with an N50 of 3,013 bp. The *R. gossypii* genome was assembled into 48 contigs with a total size of 33.334 Mb and 266× coverage, with the largest contig being 4.803 Mbp. This genome has 21.55% repeat content, composed mainly of retroelements (LINEs, Ty1/Copia, and Gypsy/DIRS1) and DNA transposons (Tc1-IS630-Pogo). BUSCO results show 98.8% completeness: of 3,786 BUSCO groups, 3,742 are complete and single copy (98.8%), 5 are fragmented (0.1%), and 39 are missing (1%) (Table 1).

**TABLE 1:** Sequencing and assembly statistics for the *Ramulariopsis gossypii* (isolate: AM24) genome

Sequencing/genome statistics	
Total read count	4,329,065
Read N50	3,013
Genome size (Mbp)	33.34
Number of contigs	48
Contig N50 (Mbp)	2.116
Mean coverage	266×
GC content (%)	50.42
Number of genes	9,707
Assembly BUSCO (dothideomycetes_odb10)	98.8
Repeat content (%)	21.55

## Data Availability

The information associated with this genome can be found at NCBI BioProject PRJNA1442825 and BioSample SAMN56709412. Raw sequence reads were submitted to the NCBI Sequence Read Archive SRR37806305. This Whole Genome Shotgun project has been deposited on the Figshare database (18).
